# Is There an Association or Not?—Investigating the Association of Depressiveness, Physical Activity, Body Composition and Sleep With Mediators of Inflammation

**DOI:** 10.3389/fpsyt.2020.00563

**Published:** 2020-06-25

**Authors:** Frank M. Schmidt, Roland Mergl, Juliane Minkwitz, Lesca M. Holdt, Daniel Teupser, Ulrich Hegerl, Hubertus Himmerich, Christian Sander

**Affiliations:** ^1^ Department of Psychiatry and Psychotherapy, University of Leipzig Medical Center, Leipzig, Germany; ^2^ Department of Clinical Psychology and Psychotherapy, Bundeswehr University Munich, Munich, Germany; ^3^ IFB Adiposity Diseases, Leipzig University Medical Center, Leipzig, Germany; ^4^ Department of Translational Research in Psychiatry, Max Planck Institute of Psychiatry (MPI), Munich, Germany; ^5^ Institute of Laboratory Medicine, University Hospital Ludwig-Maximilians-University Munich, Munich, Germany; ^6^ Department of Psychiatry, Psychosomatics and Psychotherapy, Goethe-Universität Frankfurt, Frankfurt am Main, Germany; ^7^ Department of Psychological Medicine, King’s College London, London, United Kingdom

**Keywords:** cytokines, inflammation, obesity, depression, physical activity

## Abstract

**Background:**

Cytokines are mediators of inflammation that contribute to a low-grade inflammation in different disorders like major depression and obesity. It still remains unclear which psychological and medical factors interact with cytokine regulation. In the current investigation, the association between levels of pro-and anti-inflammatory cytokines and anthropometrics, mood state (depressiveness), physical activity and sleep were investigated in a sample of community-dwelled adults.

**Methods:**

Forty-nine subjects met the inclusion criteria for analyses and were assessed at two time-points (baseline (T1) and follow-up (T2), average T1-T2-interval = 215 days). Serum cytokine measures included the pro-inflammatory cytokines interleukin (IL)-2, IL-12, IFN-γ and TNF-α, the anti-inflammatory cytokines IL-4, IL-5, IL-10 and IL-13 and the granulocyte-macrophage colony-stimulating factor (GM-CSF); anthropometrics were assessed *via* physical examination, depressiveness was assessed *via* Beck Depression Inventory (BDI)2, parameters of physical activity (steps, METs) and sleep (night/total sleep duration) were measured *via* a 1-week actigraphy.

**Results:**

Correlation analyses showed low-to moderate significant relationships between the majority of cytokines and the BDI2 at T1, positive correlation with weight and BMI at T1 and T2, and negative correlations with the number of steps and METs at T2 and T2. Regression analyses for T1 revealed that the BDI2 score was the best positive predictor for the concentrations of all nine cytokines, followed by the number of steps and the nightsleep duration as negative predictors. At T2, the amount of steps was found to be negatively associated with IL-4, IL5, IL-10, GM-CSF, IFN-γ, and TNF-α, whereas the BMI could significantly predict IL-12 and IL-13. The BDI2-score was not significantly associated with any of the cytokines. No associations could be found between dynamics in cytokines from T1 and T2 and changes in any of the variables.

**Discussion:**

The present results indicate an influence of physical activity, subjective well-being and body composition on inflammatory mediators. Since there was no standardized intervention targeting the independent variables between T1 and T2, no assumptions on causality can be drawn from the association results.

## Introduction

Cytokines are a category of heterogeneous peptides produced by various cells, such as macrophages, T lymphocytes, B lymphocytes and mast cells, which are critically involved in cell signaling and immune response. Previously, it could be demonstrated that both, subjects suffering from major depression and subjects with obesity show elevations in their cytokine profiles (1–5). This repeated finding leads to the assumption that both disorders are characterized by a low-grade inflammation which may be a common pathway that could explain the high comorbidity rates between the two disorders (6, 7). Concentrations in inflammatory cytokines may further relate to the degree of obesity (1, 8, 9), whereas results on the association of cytokines with the severity of depression or certain subtypes of depression are still conflicting (10–14). Whereas pharmaco-psychiatric treatment has not yet proven to have a consistent effect upon cytokine regulation that accompanies the therapeutic course (10, 15, 16), some non-pharmacological interventions, such as regular exercise, are effective in treating both depression and obesity and could have anti-inflammatory properties (17–19). Further, sleep properties, which are affected in depression and obesity as well as their related disorders like sleep apnea and fatigue, are discussed to interact with immune regulation (20, 21).

Although a lot of research has been performed to shed light on the multilateral facets of cytokines, and meta-analyses confirm certain assumptions [e.g. elevations of cytokines in depression (3, 4)], there are high variances of cytokine values within and between samples. The majority of investigations have been performed using a cross-sectional approach, and only few replicated the findings in the same sample or in a longitudinal design. Hence, the impact of different social, psychological and medical factors on cytokines and vice versa is yet far from clear (22).

To shed light on different factors that may be related to cytokine regulation, the associations between nine different pro- and anti-inflammatory cytokines and anthropometrics, mood states (depressiveness), physical activity, energy expenditure and parameters of sleep were examined in a sample of community-dwelled adults at two different time points. We further aimed to investigate if dynamics in these parameters between the two measurements were associated with changes in cytokine levels.

## Materials and Methods

For the present study, data were used from the OBDEP sub-project (Obesity and Depression: pathogenic role of sleep and wakefulness regulation, motor activity level and neurochemical aspects), which was conducted at the Department of Psychiatry and Psychotherapy of the University Hospital Leipzig within the framework of the Integrated Research and Treatment Center for Adiposity Diseases Leipzig (IFB Adiposity). The study was approved by the Leipzig University Ethics Committee (#015–10–18012009). All participants were aged 18 to 70 years and gave written informed consent. The OBDEP project comprised of two assessment points (baseline and follow-up), in which participants were requested to fill out questionnaires related to mood and diet (including the German version of the revised Beck Depression Inventory, second edition (BDI2) (23, 24), to give a blood sample for cytokine analysis and wore an actigraphic device for one week to assess physical activity and sleep (see below). No systematic intervention took place between the two assessments, apart from many of the obese participants receiving information on healthy nutrition and physical activity while waiting for an appointment for a stomach reduction. Data from the baseline assessment has been published elsewhere (1, 2, 11, 25, 26), while results from the follow-up assessment have not yet been presented.

### Sample

In total, 304 participants had been recruited for the OBDEP project either from the outpatient clinic of the IFB Adiposity, from the Department of Psychiatry and Psychotherapy of the University Hospital Leipzig and *via* announcements [details on the recruitment and pre-screening process can be found elsewhere (1, 2)]. Eligible participants were then invited to the study center, where inclusion criteria were assessed in more detail by a study physician. Assessments for current and past history of physical and mental health problems as well as current medication were performed using standardized forms. Reasons for non-inclusion were acute or chronic infections, current medication with a recognized major impact on the immune system, current psychiatric medication, psychiatric and neurological disorders apart from depression, and a history of head injury with loss of consciousness exceeding 1 h.

Participants included into the OBDEP project underwent a full physical examination (including blood sampling) by qualified healthcare professionals. Weight [kg] was determined in underwear and without shoes using a digital scale calibrated and standardized using a weight of known mass. Height [cm] was recorded using a stadiometer with participants standing on a flat surface at a right angle to the vertical board of the stadiometer. BMI [kg/m^2^] was defined as body weight [kg] divided by the square of height [m^2^].

For the present study, we selected all participants who had partaken in the optional follow-up assessment (N=107). Since only 35% of the original sample took part in the follow-up assessment we compared those subjects with the non-participants. There were no statistical differences concerning sex (62% versus 66% females, p=0.401), but those subjects completing the follow-up were significantly older (41.5 versus 38.2 years, p=0.032) and had a lower T1-BMI (34.0 versus 37.2, p=0.027). We then excluded those subjects for whom at least one of the following exclusion criteria was present: a) retest interval < 150 or > 300 days (N=4); b) cytokine levels had not been measured at both assessments (N=6); c) missing BMI data (N=5); d) missing data in BDI2 questionnaires (N=10) or e) actigraphy data not fulfilling analysis quality criteria (see below) (N=36). In addition, two subjects were excluded post-hoc from the final sample because the levels of the majority of cytokines were rated as extreme outliers (> 2 standard deviations compared to the overall group average, which could indicate a current illness). In total, 49 participants were included in the subsequent analyses. When comparing the included with the excluded participants, there was a trend for differences concerning sex (53% versus 69% females, p=.092), but no statistical differences in age (41.7 versus 41.4 years, p=.925) or T1-BMI (33.4 versus 34.5, p=.607).

### Cytokine Measurements

Immediately after blood drawing, serum probes were centrifuged at 3,000 rpm for 10 min. The supernatant was aliquoted and stored in non-absorbing polypropylene tubes of 300 ml, which were subsequently snap-frozen in liquid N2 and stored in freezers at −80°C until further measurement. Cytokines were measured at the Institute of Laboratory Medicine of the University Hospital of the Ludwig-Maximilians-University Munich using the Bio-Plex Pro™ human cytokine Th1/Th2 immunoassay (Bio Rad, Germany), a 96-well kit that includes coupled magnetic beads and detection antibodies. This multiplex assay detects pro-inflammatory IL-2, IL-12, GM-CSF, IFN-γ, TNF-α and anti-inflammatory IL-4, IL-5, IL-10, IL-13. The intraassay coefficient of variation (CV) for cytokines was between 1.6% and 3.8%. If cytokine levels were lower than the detection cut-off of the immunoassay (< OOR), there were assigned a value of 0.

### Actigraphy

On both occasions (baseline and follow-up), a 1-week actigraphy recording was performed, using the SenseWear^®^ Pro 3 actigraph (SWA; BodyMedia Inc.; Pittsburgh, Pennsylvania). The SWA is attached to the upper right arm and records 2-axis body acceleration, skin temperature, heat flux and galvanic skin response. Furthermore, SWA detects periods in which it is not worn (off-arm periods). Actigraphic data were analyzed using SenseWear^®^ Professional Software Version 7 (BodyMedia Inc.). Based on validated proprietary scoring algorithms included in the software, each minute of the recorded data is scored as laying down [yes/no] or sleep [yes/no]. Furthermore, amount of steps and MET levels are given for each 1-min timeframe. Several studies have demonstrated that the SWA provides accurate estimates of energy expenditure during rest and daily life activities, comparable to the gold standards of indirect calorimetry and doubly labeled water (27–33).

Scored data were entered into a customized Excel-Template for further data preparation. Participants had kept a sleep and activity diary throughout the recording period and according to the respective information provided by the participants, nightsleep intervals (NSI) was estimated (ranging from first minute to last minute scored as laying down in close proximity to the noted bed times within the sleep diary). Accordingly, the daytime interval (DTI) was determined as duration between two consecutive NSI. Sleep duration (SD) was calculated for each NSI and each DTI (=sum of minutes scored as sleep within the NSI or DTI). Total sleep duration (TSD) was calculated by adding night sleep duration within one NSI (e.g. nightsleep from Friday to Saturday) with daytime sleep duration of the following DTI (e.g. daysleep on Saturday). In addition, for each DTI, the variables *steps* (=sum of steps per 1 min-segment) and *METs* (=mean of all MET values per 1 min-segment) were calculated. Afterwards daily values of these four measures were averaged to obtain mean values for the total week. Datasets were only included in the analysis if the averaged values comprised of at least 4 NSI/DTI-cycles, respectively, with at least one NSI/DTI-cycle during the weekend.

### Statistical Analysis

The Kolmogorov-Smirnov (K-S) test was used to examine whether the cytokine levels were normally distributed or not. Therefore, in a first step, outliers cases (± 2 standard deviations) were detected and removed from the respective analysis. Since most outliers (e.g. 9 out of 10 outliers at T2) could be attributed to two specific participants, these two participants were post-hoc excluded from all analyses. After outlier removal, the K-S test still attested for non-Gaussian distribution, therefore cytokine values were normalized using a square root (SQR) or logarithmic (LN) transformation. Based on K-S results, the best transformation was determined for each cytokine level (SQR transformation: IL-2, IL-4, IL-10, IL-12, IFN-γ, TNF-α; LN transformation: IL-5, IL-13, GM-CSF).

The association between cytokine levels (response variables) and anthropometric, psychometric and actigraphic parameters (independent variables) were tested using multiple linear regression (MLR) models. The initial MLR models included all independent variables. A stepwise backwards linear regression method was used: the variables which contributed least to the aforementioned model had been removed in subsequent steps until only statistically significant variables remained (p < 0.05). In order to assess multicollinearity of the independent variables, tolerance statistics like the variance inflating factor were also calculated.

Changes between baseline (T1) and follow-up (T2) were quantified by calculation difference scores (Δ=T2-T1). The K-S test showed a Gaussian distribution in all cytokine change scores but Δ IL-5. This non-Gaussian distribution could be attributed to one outlier case which was therefore excluded from the respective MLR analysis.

For correlation analyses of cytokine levels and independent variables, Spearman rank correlation coefficients were calculated, since most independent variables also exhibited a non-Gaussian distribution. Therefore, non-transformed cytokine levels were used for all correlation analyses. For similar reasons, differences between T1 and T2 values were tested using the Wilcoxon test. All analyses were conducted by using the statistical software SPSS 23 (IBM SPSS Statistics for Windows, version 23 (IBM Corp., Armonk, N.Y., USA)). Also, the significance level was taken to be α=0.05 (two-tailed).

## Results

Forty-nine participants were included in the final analyses in this study (N=23 males (46.9%), mean age 41.64 years (SD=11.8 years, range 22–62 years). On average, the follow-up assessment (T2) of the included participants was performed 215.76 days (SD=27.24, range 170–288) after their baseline assessment (T1).

Results concerning the anthropometrics (weight, BMI), psychometric test (BDI2 score), and actigraphy results (night sleep duration, total sleep duration, as well as number of steps and average METs during the wake phase) measured at T1 and T2 are depicted in **Table 1A**. On group level, the Wilcoxon Test revealed no significant differences between T1 and T2 in all the possible predictors for cytokine changes. Cytokine levels at T1 and T2 are shown in **Table 1B**. On average, there was a numerical decrease in serum levels of all cytokines, but according to the Wilcoxon test, this decrease was only significant for IL-12, with a trend for IL-2 and IL-13.

**Table 1A:** Comparison of baseline (T1) and follow-up (T2) values of anthropometrics, psychometric and actigraphic parameters.

	Baseline (T1)				Follow up (T2)				Wilcoxon
	Mean	SD	Min	Max	Mean	SD	Min	Max	Z/p
**Weight (in kg)**	99.2	36.25	52	179	97.4	33.57	53	170	−0.527/0.598
**BMI (kg/m^2^)**	33.4	11.15	18.3	61.4	32.7	10.31	18.9	61.3	−0.954/0.340
**BDI2-Score**	6.4	6.68	0	25	6.7	8.06	0	31	−0.350/0.726
**Nightsleep Duration (hh:mm)**	5:53	1:03	2:53	7:56	6:01	1:02	3:51	7:45	−0.741/0.459
**Total Sleep Duration (hh:mm)**	6:18	1:08	3:13	8:23	6:23	1:02	4:00	8:13	−0.453/0.651
**Steps**	10087.1	4891.49	1787.6	21407.3	9725.7	4259.84	2355.5	18357.67	−0.831/0.406
**METs**	1.68	0.56	0.83	3.02	1:62	0.42	0.93	2.54	−1.457/0.145

**Table 1B:** Comparison of cytokine levels at baseline (T1) and follow-up (T2).

	Baseline (T1)				Follow up (T2)				Wilcoxon
	Mean	SD	Min	Max	Mean	SD	Min	Max	Z/p
**Pro-inflammatory**									
**IL-2 [pg/mL]**	7.16	7.11	0.00	25.96	5.76	5.62	0.00	18.19	−1.885/0.059
**IL-12 [pg/mL]**	10.33	8.33	0.00	40.90	8.79	6.79	0.00	31.96	**−2.072/0.038**
**GM-CSF [pg/mL]**	38.03	20.91	3.83	97.78	35.02	16.05	9.10	65.55	−1.323/0.186
**IFN-γ [pg/mL]**	121.19	64.64	0.00	280.09	112.85	57.15	19.44	269.73	−1.169/0.242
**TNF-α [pg/mL]**	31.19	18.73	3.71	84.87	28.21	14.78	6.34	64.95	−1.323/0.186
**Anti-inflammatory**									
**IL-4 [pg/mL]**	4.56	2.57	0.09	10.32	4.13	2.10	0.79	9.84	−1.263/0.206
**IL-10 [pg/mL]**	4.02	3.09	0.00	18.03	3.60	3.79	0.00	18.22	−0.850/0.395
**IL-5 [pg/mL]**	3.02	2.53	0.00	13.10	2.56	1.62	0.00	6.87	−1.550/0.121
**IL-13 [pg/mL]**	5.55	4.98	0.00	18.16	4.59	3.87	0.00	13.90	−1.707/0.088

In bold: p > 0.05.

Correlations between cytokine levels and BMI, BDI2 score and actigraphy parameters at T1 are depicted in **Table 2A**. Weight showed significant but weak associations with IL-2, IL-10, IL-12 and IL-13. BMI had still weak but slightly stronger significant associations with IL-2, IL-10, IL-12, IL-13 and also TNF-α. The BDI2-scores were positively associated with all cytokine levels except IL-4 and IL-5. Steps had moderate, negative associations with all cytokine levels apart from the growth factor GM-CSF. METs only showed significant, moderate negative associations with IL-2, IL-10, IL-12, and IL-13. For night and total sleep duration, none of the negative associations reached significance.

**Table 2A:** Spearman Rank correlation coefficients between cytokine levels and antropometric, psychometric and actigraphic parameters at T1.

	Pro-inflammatory					Anti-inflammatory			
	IL-2	IL-12	GM-CSF	IFN-γ	TNF-α	IL-4	IL-5	IL-10	IL-13
**Weight**	0.326*	0.387**	0.224	0.273	0.278	0.248	0.277	0.371*	0.387**
**BMI**	0.339*	0.423**	0.236	0.277	0.289*	0.245	0.277	0.409**	0.423**
**BDI2 Score**	0.313*	0.330*	0.322*	0.311*	0.348*	0.281	0.278	0.334*	0.330*
**Night Sleep Duration**	−0.251	−0.161	−0.199	−0.227	−0.223	−0.214	−0.197	−0.260	−0.161
**Total Sleep Duration**	−0.244	−0.171	−0.232	−0.260	−0.219	−0.218	−0.184	−0.260	−0.171
**Steps**	−0.375**	−0.317*	−0.264	−0.298*	−0.350*	−0.299*	−0.305*	−0.388**	−0.317*
**METs**	−0.330*	−0.307*	−0.203	−0.279	−0.273	−0.252	−0.192	−0.375**	−0.307*

Annotations: * = p < 0.05; ** = p < 0.01.

Correlations between cytokine levels and BMI, BDI2 score and actigraphy parameters at T2 are depicted in **Table 2B**. Weight and BMI showed significant and weak associations with all cytokines that were stronger in all cases than what had been found at T1. On the other hand, steps and METs showed significant negative associations with all cytokine levels that were also stronger than those that had been found at T1. No associations with any cytokine level were found for BDI2, night and total sleep duration. Steps had moderate, negative associations with all cytokine levels except for GM-CSF. METs showed moderate, significant, negative associations with IL-2, IL-10, IL-12, and IL-13.

**Table 2B:** Spearman Rank correlation coefficients between cytokine levels and antropometric, psychometric and actigraphic parameters at T2.

	Pro-inflammatory					Anti-inflammatory			
	IL-2	IL-12	GM-CSF	IFN-γ	TNF-α	IL-4	IL-5	IL-10	IL-13
**Weight**	0.484**	0.494**	0.348*	0.387**	0.415**	0.373**	0.394**	0.431**	0.407**
**BMI**	0.475**	0.479**	0.352*	0.377**	0.402**	0.361*	0.363*	0.428**	0.427**
**BDI2 Score**	0.075	0.154	0.029	0.025	0.058	−0.014	0.110	0.119	−0.007
**Night Sleep Duration**	−0.027	−0.118	0.006	−0.038	−0.030	−0.057	−0.037	−0.086	−0.092
**Total Sleep Duration**	−0.027	−0.094	−0.013	−0.062	−0.023	−0.072	−0.056	−0.083	−0.022
**Steps**	−0.517**	−0.408**	−0.438**	−0.478**	−0.497**	−0.453**	−0.486**	−0.499**	−0.317*
**METs**	−0.508**	−0.442**	−0.413**	−0.483**	−0.471**	−0.447**	−0.432**	−0.459**	−0.348*

Annotations: * = p < 0.05; ** = p < 0.01.

When changes in cytokine levels between T1 and T2 were correlated with changes in the independent variables (see **Table 2C**), none of the associations reached significance except for a weak negative association between changes in night sleep duration and changes in levels of TNF-α.

**Table 2C:** Spearman Rank correlation coefficients between difference values (T2-T1) in cytokine levels and antropometric, psychometric and actigraphic parameters.

	Pro-inflammatory					Anti-inflammatory			
	Δ IL-2	Δ IL-12	Δ GM-CSF	Δ IFN-γ	Δ TNF-α	Δ IL-4	Δ IL-5	Δ IL-10	Δ IL-13
Δ **Weight**	−0.153	−0.124	−0.077	−0.035	−0.096	−0.030	−0.051	−0.113	−0.201
Δ **BMI**	−0.103	−0.061	−0.038	−0.003	−0.048	0.023	0.017	−0.050	−0.122
Δ **BDI2 Score**	−0.021	−0.073	0.000	0.004	0.040	−0.016	−0.009	−0.066	−0.069
Δ **Night Sleep Duration**	−0.155	−0.234	−0.219	−0.222	−0.284*	−0.236	−0.209	−0.072	−0.094
Δ **Total Sleep Duration**	−0.162	−0.262	−0.205	−0.245	−0.267	−0.210	−0.208	−0.058	−0.108
Δ **Steps**	0.066	0.177	0.060	−0.003	−0.039	0.077	0.045	0.004	0.184
Δ **METs**	0.079	0.184	0.045	0.022	−0.032	0.067	0.031	0.086	0.190

Annotations: * = p < 0.05; ** = p < 0.01.

The relationship between cytokine levels and anthropometric, psychometric and actigraphic parameters (independent variables) were tested using multiple linear regression (MLR) models. As expected, several of the independent variables highly correlated (weight/BMI: rho_T1_ = 0.954, rho_T2_ = 0.950, rho_Δ_=0.961; night/total sleep duration: rho_T1_ = 0.934, rho_T2_ = 0.908, rho_Δ_=0.899; steps/METs: rho_T1_ = 0.861, rho_T2_ = 0.821, rho_Δ_=0.659). To avoid multicollinearity, only 4 independent variables (BMI, BDI2 scores, night sleep duration, and step counts during wake phase) were included in the initial MLR models.

Findings regarding initial as well as final MLR models for cytokine levels at baseline (T1) are summarized in **Table 3A**. The final MLR models demonstrated a significantly positive association of the BDI2 sum scores with cytokine serum levels for IL-2 (p=0.049), IL-4 (p=0.036), IL-5 (p=0.039), Il-12 (p=0.013), Il-13 (p=0.005), GM-CSF (p=0.022), INF-γ (p=0.024) and TNF-α (p=0.031). Moreover, there was a significantly negative association between the number of steps with cytokine levels regarding IL-2 (p=0.031), IL-10 (p=0.023) and TNF-α (p=0.029) and a significantly negative association of night sleep duration with IL-12 levels (p=0.034).

**Table 3A:** Regression coefficients of cytokine levels on the predictor variables recorded at baseline (T1).

Predictors	Primary Model (Include)					Final Model (stepwise backwards)				
	Beta	CI^−^	CI^+^	P-Value	Stand. Coeff.	Beta	CI^−^	CI^+^	P-Value	Stand. Coeff.
IL-2 (SQR-trans.)	Model 1: N = 49/R^2^ =.263/corr. R^2^^=^ 0.196					Model 3: R^2^ = 0.216/corr. R^2^ = 0.182				
BMI	−0.011	−0.059	0.037	0.643	−0.097					
BDI2-Score	0.057	−0.004	0.117	0.065	0.297	0.053	0.000	0.105	0.049	0.275
Nightsleep Duration	−0.275	−0.607	0.056	0.101	−0.228					
Steps	−8.2E-5	−1.8E-4	1.0E-4	0.081	−0.315	−7.9E-5	−1.5E-4	−0.8E-5	0.031	−0.304
IL-12 (SQR-trans.)	Model 1: N = 49/R^2^ = 0.224/corr. R^2^ = 0.153					Model 3: R^2^ = 0.222/corr. R^2^ = 0.188				
BMI	−0.001	−0.048	0.047	0.982	−0.005					
BDI2-Score	0.062	0.001	0.122	0.046	0.331	0.063	0.014	0.112	0.013	0.340
Nightsleep Duration	−0.330	−0.661	0.001	0.051	−0.280	−0.337	−0.647	−0.027	0.034	−0.287
Steps	−1.0E-5	−1.0E-4	8.2E-5	0.821	−0.041					
GMCSF (LGn-trans.)	Model 1: N = 49/R^2^ = 0.193/corr. R^2^ = 0.119					Model 4: R^2^ = 0.107/corr. R^2^ = 0.088				
BMI	−0.011	−0.035	0.012	0.342	−0.209					
BDI2-Score	0.030	0.000	0.060	0.049	0.333	0.030	0.005	0.055	0.022	0.327
Nightsleep Duration	−0.110	−0.275	0.055	0.185	−0.192					
Steps	−3.7E-5			0.112	−0.298					
IFN-γ (SQR-trans.)	Model 1: N = 49/R^2^ = 0.222/corr. R^2^ = 0.151					Model 3: R^2^ = 0.177/corr. R^2^ = 0.142				
BMI	−0.037	−0.158	0.038	0.539	−0.132					
BDI2-Score	0.146	−0.007	0.298	0.060	0.311	0.148	0.020	0.275	0.024	0.315
Nightsleep Duration	−0.666	−1.500	0.168	0.114	−0.225	−0.717	−1.520	0.086	0.079	−0.242
Steps	−1.7E-4	−4.1E-4	5.6E-5	0.134	−0.276					
TNF-α (SQR-trans.)	Model 1: N = 49/R^2^ = 0.288/corr. R^2^ = 0.224					Model 3: R^2^ = 0.233/corr. R^2^ = 0.200				
BMI	−0.024	−0.085	0.037	0.429	−0.163					
BDI2-Score	0.086	0.009	0.163	0.030	0.346	0.074	0.007	0.141	0.031	0.300
Nightsleep Duration	−0.375	−0.797	0.046	0.080	−0.240					
Steps	−1.2E-4	−2.4E-4	9.9E-8	0.050	−0.348	−1.0E-4	−1.9E-4	−1.1E-5	0.029	−0.303
IL-4 (SQR-trans.)	Model 1: N = 49/R^2^ = 0.223/corr. R^2^ = 0.152					Model 3: R^2^ = 0.175/corr. R^2^ = 0.139				
BMI	−0.008	−0.029	0.014	0.478	−0.152					
BDI2-Score	0.024	−0.002	0.051	0.074	0.295	0.024	0.002	0.047	0.036	0.292
Nightsleep Duration	−0.130	−0.278	0.017	0.082	−0.249	−0.138	−0.281	0.004	0.057	−0.264
Steps	−3.3E-5	−7.4E-5	0.8E-5	0.114	−0.291					
IL-5 (LGn-trans.)	Model 1: N = 49/R^2^ = 0.151/corr. R^2^ = 0.073					Model 4: R^2^ = 0.087/corr. R^2^ = 0.068				
BMI	−0.005	−0.030	0.019	0.659	−0.099					
BDI2-Score	0.025	−0.006	0.056	0.110	0.275	0.027	0.001	0.053	0.039	0.295
Nightsleep Duration	−0.124	−0.294	0.046	0.148	−0.215					
Steps	−2.1E-5			0.383	−0.166					
IL-10 (SQR-trans.)	Model 1: N = 49/R^2^ = 0.245/corr. R^2^ = 0.176					Model 3: R^2^ = 0.205/corr. R^2^ = 0.171				
BMI	−0.007	−0.042	0.028	0.677	−0.088					
BDI2-Score	0.036	−0.009	0.080	0.113	0.257	0.033	−0.005	0.071	0.090	0.238
Nightsleep Duration	−0.182	−0.426	0.061	0.138	−0.208					
Steps	−6.3E-5	−1.3E-4	0.5E-5	0.068	−0.333	−6.1E-5	−1.1E-4	−0.9E-5	0.023	−0.324
IL-13 (LGn-trans.)	Model 1: N = 49/R^2^ = 0.217/corr. R^2^ = 0.146					Model 4: R^2^ = 0.155/corr. R^2^ = 0.137				
BMI	0.007	−0.027	0.040	0.693	0.085					
BDI2-Score	0.037	−0.004	0.079	0.078	0.292	0.050	0.016	0.085	0.005	0.393
Nightsleep Duration	−0.116	−0.345	0.113	0.314	−0.143					
Steps	−2.2E-5	−8.6E-5	4.2E-5	0.490	−0.126					

Results of the (initial and final) MLR models for cytokine levels at T2 are presented in **Table 3B**. The final MLR models showed a significant negative association of number of steps during the wake phase with cytokine serum levels for IL-2 (p < 0.001), IL-4 (p=0.002), IL-5 (p=0.001), IL-10 (p < 0.001), GM-CSF (p=0.003), IFN-γ (p=0.002) and TNF-α (p < 0.001). A significant positive association with the BMI was found for IL-12 (p=0.009) and IL-13 (p=0.007).

**Table 3B:** Regression coefficients of cytokine levels on the predictor variables recorded at follow-up (T2).

Predictors	Primary Model (Include)					Final Model (stepwise backwards)				
	Beta	CI^−^	CI^+^	P-Value	Stand. Coeff.	Beta	CI^−^	CI^+^	P-Value	Stand. Coeff.
IL-2 (SQR-trans.)	Model 1: N = 49/R^2^ = 0.340/corr. R^2^ = 0.281					Model 3: R^2^ = 0.314/corr. R^2^ = 0.284				
BMI	0.036	−0.001	0.074	0.059	0.345	0.029	−0.003	0.061	0.079	0.271
BDI2-Score	−0.008	−0.047	0.031	0.677	−0.060					
Nightsleep Duration	0.169	−0.103	0.440	0.216	−0.162					
Steps	9.2E-5	−1.7E-4	−1.1E-5	0.027	−0.359	−9.1E-5	−1.7E-4	−1.3E-5	< 0.001	−0.356
IL-12 (SQR-trans.)	Model 1: N = 49/R^2^ = 0.170/corr. R^2^ = 0.095					Model 4: R^2^ = 0.137/corr. R^2^ = 0.118				
BMI	0.019	−0.022	0.060	0.355	0.187	0.038	0.010	0.065	0.009	0.370
BDI2-Score	0.015	0.027	0.057	0.483	0.113					
Nightsleep Duration	0.000	−0.293	0.294	0.999	0.000					
Steps	−5.5E-5	−1.4E-4	3.3E-5	0.213	−0.223					
GM-CSF (LN-trans.)	Model 1: N = 49/R^2^ = 0.198/corr. R^2^ = 0.169					Model 4: R^2^ = 0.169/corr. R^2^ = 0.151				
BMI	0.008	−0.010	0.026	0.394	0.169					
BDI2-Score	−0.007	−0.025	0.012	0.455	−0.118					
Nightsleep Duration	0.060	−0.069	0.189	0.355	0.133					
Steps	−3.9E-5	−7.8E-5	−7.9E-7	0.046	−0.356	−4.528	−7.5E-5	−1.6E-5	0.003	−0.411
IFN-γ (SQR-trans.)	Model 1: N = 49/R^2^ = 0.204/corr. R^2^ = 0.132					Model 4: R^2^ = 0.189/corr. R^2^ = 0.171				
BMI	0.042	−0.058	0.143	0.401	0.166					
BDI2-Score	−0.30	−0.133	0.074	0.566	−0.091					
Nightsleep Duration	0.149	−0.575	0.873	0.681	0.059					
Steps	−2.2E-4	−4.4E-4	−0.8E-5	0.043	−0.360	−2.7E-4	−4.3E-4	−1.1E-4	0.002	−0.434
TNF-α (SQR-trans.)	Model 1: N = 49/R^2^ = 0.283/corr. R^2^ = 0.217					Model 4: R^2^ = 0.250/corr. R^2^ = 0.234				
BMI	0.028	−0.021	0.077	0.261	0.211					
BDI2-Score	−0.016	−0.067	0.034	0.512	−0.098					
Nightsleep Duration	0.171	−0.180	0.522	0.331	0.132					
Steps	−1.3E-4	−2.4E-4	−2.8E-5	0.014	−0.419	−1.5E-4	−2.4E-4	−7.8E-5	< 0.001	−0.500
IL-4 (SQR-trans.)	Model 1: N = 49/R^2^ = 0.204/corr. R^2^ = 0.132					Model 4: R^2^ = 0.185/corr. R^2^ = 0.168				
BMI	0.007	−0.010	0.024	0.410	0.163					
BDI2-Score	−0.006	−0.024	0.011	0.487	−0.110					
Nightsleep Duration	0.036	−0.087	0.159	0.560	0.083					
Steps	−3.9E-5	−7.5E-5	−0.2E-5	0.040	−0.366	−4.5E-5	−7.3E-5	−1.7E-5	0.002	−0.430
IL-5 (LN-trans.)	Model 1: N = 49/R^2^ = 0.220/corr. R^2^ = 0.149					Model 4: R^2^ = 0.205/corr. R^2^ = 0.188				
BMI	0.005	−0.013	0.023	0.589	0.105					
BDI2-Score	0.000	−0.019	0.018	0.965	−0.007					
Nightsleep Duration	0.052	−0.078	0.181	0.424	0.113					
Steps	−4.7E-5	−8.6E-5	−0.9E-5	0.018	−0.420	−5.1E-5	−8.0E-5	−2.1E-5	0.001	−0.453
IL-10 (SQR-trans.)	Model 1: N = 49/R^2^ = 0.251/corr. R^2^ = 0.183					Model 4: R^2^ = 0.236/corr. R^2^ = 0.220				
BMI	0.015	−0.017	0.046	0.352	0.178					
BDI2-Score	−0.006	−0.038	0.026	0.716	−0.056					
Nightsleep Duration	0.024	−0.200	0.249	0.828	0.030					
Steps	−7.8E-5	−1.5E-4	−1.1E-5	0.023	−0.394	−9.6E-5	−1.5E-4	−4.5E-5	< 0.001	−0.486
IL-13 (LN-trans.)	Model 1: N = 49/R^2^ = 0.178/corr. R^2^ = 0.103					Model 4: R^2^ = 0.145/corr. R^2^ = 0.127				
BMI	0.038	0.006	0.070	0.021	0.475	0.031	0.009	0.052	0.007	0.381
BDI2-Score	−0.021	−0.054	0.012	0.210	−0.202					
Nightsleep Duration	0.004	−0.226	0.234	0.971	0.005					
Steps	−4.6E-7	−6.9E-5	6.8E-5	0.989	−0.002					

MLR models for changes in cytokine levels (T2–T1) are presented in **Table 3C**. Out of all the independent variables, only changes in night sleep duration seemed to be somewhat negatively associated with changes in cytokine levels, however, this effect showed only a statistical tendency in the final models for IL-4 (p=0.100), IFN-γ (p=0.098) and TNF-α (p=0.085).

**Table 3C:** Regression coefficients of changes in cytokine levels (T2-T1) on the changes of predictor variables.

Psredictor	Primary Model (Include)					Final Model (stepwise backwards)				
	Beta	CI^−^	CI^+^	P-Value	Stand. Coeff.	Beta	CI^−^	CI^+^	P-Value	Stand. Coeff.
Δ IL−2	Model 1: N = 49/R^2^ = 0.045/corr. R^2^ = −0.041					Model 5: R^2^ = 0.000/corr. R^2^ = 0.000				
Δ BMI	−0.326	−1.014	0.362	0.345	−0.147					
Δ BDI2-Score	0.029	−0.203	0.260	0.802	0.038					
Δ Night SD	−0.900	−2.489	0.689	0.260	−0.174					
Δ Steps	−1.5E-4	−0.001	3.1E-4	0.517	−0.099					
Δ IL-12	Model 1: N = 49/R^2^ = 0.045/corr. R^2^ = −0.042					Model 5: R^2^ = 0.000/corr. R^2^ = 0.000				
Δ BMI	−0.029	−0.729	0.671	0.934	−0.013					
Δ BDI2-Score	0.006	−0.230	0.241	0.962	0.007					
Δ Night SD	−1.027	−2.644	0.591	0.208	−0.195					
Δ Steps	1.1E-4	−3.7E-4	0.001	0.650	0.069					
Δ GM-CSF	Model 1: N = 49/R^2^ = 0.056/corr. R^2^ = −0.029					Model 5: R^2^ = 0.000/corr. R^2^ = 0.000				
Δ BMI	−0.419	−2.727	1.889	0.716	−0.056					
Δ BDI2-Score	0.078	−0.699	0.855	0.841	0.030					
Δ Night SD	−4.194	−9.525	1.138	0.120	−0.240					
Δ Steps	−2.0E-4	−0.002	0.001	0.799	−0.039					
Δ IFN-γ	Model 1: N = 49/R^2^ = 0.064/corr. R^2^ = −0.021					Model 4: R^2^ = 0.057/corr. R^2^ = 0.037				
Δ BMI	−0.116	−7.485	7.253	0.975	−0.005					
Δ BDI2-Score	0.042	−2.439	2.552	0.973	0.005					
Δ Night SD	−13.859	−30.882	3.164	0.108	−0.247	−13.371	−29.327	2.586	0.098	−0.239
Δ Steps	−0.001	−0.006	0.004	0.571	−0.086					
Δ TNF-α	Model 1: N = 48/R^2^ = 0.091/corr. R^2^ = 0.009					Model 4: R^2^ = 0.062/corr. R^2^ = 0.042				
Δ BMI	−0.557	−2.523	1.409	0.571	−0.086					
Δ BDI2-Score	0.144	−0.518	0.806	0.663	0.064					
Δ Night SD	−4.153	−8.695	0.389	0.072	−0.274	−3.768	−8.078	0.542	0.085	−0.248
Δ Steps	−0.001	−0.002	0.001	0.322	−0.148					
Δ IL-4	Model 1: N = 49/R^2^ = 0.061/corr. R^2^ = −0.025					Model 4: R^2^ = 0.057/corr. R^2^ = 0.037				
Δ BMI	−0.054	−0.354	0.246	0.720	−0.055					
Δ BDI2-Score	0.004	−0.097	0.105	0.935	0.012					
Δ Night SD	−0.573	−1.266	0.120	0.102	−0.252	−0.541	−1.190	0.107	0.100	−0.238
Δ Steps	−2.8E-5	−2.3E-4	1.7E-4	0.779	−0.042					
Δ IL-5	Model 1: N = 48/R^2^ = 0.024/corr. R^2^ = −0.067					Model 5: R^2^ = 0.000/corr. R^2^ = 0.000				
Δ BMI	−0.012	−0.206	0.182	0.904	−0.019					
Δ BDI2-Score	0.003	−0.062	0.068	0.929	0.014					
Δ Night SD	−0.201	−0.649	0.247	0.370	−0.141					
Δ Steps	−3.4E-5	−1.7E-4	1.0E-4	0.616	−0.078					
Δ IL-10	Model 1: N = 49/R^2^ = 0.016/corr. R^2^ = −0.073					Model 5: R^2^ = 0.000/corr. R^2^ = 0.000				
Δ BMI	−0.002	−0.371	0.367	0.992	−0.002					
Δ BDI2-Score	0.004	−0.120	0.128	0.948	0.010					
Δ Night SD	−0.288	−1.140	0.564	0.500	−0.105					
Δ Steps	−6.3E-5	−3.1E-4	1.9E-4	0.614	−0.078					
Δ IL-13	Model 1: N = 49/R^2^ = 0.032/corr. R^2^ = −0.056					Model 5: R^2^ = 0.000/corr. R^2^ = 0.000				
Δ BMI	−0.096	−0.540	0.349	0.667	−0.067					
Δ BDI2-Score	0.030	−0.120	0.179	0.692	0.060					
Δ Night SD	−0.334	−1.361	0.693	0.516	−0.101					
Δ Steps	1.1E-4	−1.9E-4	4.1E-4	0.450	0.116					

## Discussion

In this investigation, we examined the impact of the four factors BMI, depressiveness, physical activity, and sleep duration on the serum levels of nine different pro- and anti-inflammatory cytokines in a sample of 49 community-dwelled subjects at two different time points. Further, we investigated if dynamics in any of the variables between the two time points were accountable for changes in cytokine levels.

Regarding the correlation analyses, the majority of cytokines were found to weakly correlate with the BMI and weight in a positive direction at both time points. The number of steps and METs correlated in a negative direction at both T1 and T2. The results for the BDI2, however, were inconsistent with significant weak correlations at T1 which were absent at T2. In the regression analyses, the physical activity could explain IL-2, IL-10, and TNF-α at T1 and was negatively involved in all cytokines except IL-12 and IL-13 at T2. On the other hand, the mood state assessed with the BDI2 showed a disparity between the two cross-sectional time points, as the significant positive association with all cytokines at T1 could not be repeated for any of the cytokines in the follow-up assessment. For all analyses, the direction of the association of the parameters did not depend upon the pro-or anti-inflammatory characteristics of the cytokines. Concerning changes in any of the four factors included into the analyses, none was found associated with dynamics in cytokines from T1 and T2.

Although no conclusions on causality can be drawn from association studies, our results still support the assumption that physical activity could influence mediators of inflammation (17, 18) and validated previous findings of a disparity in cytokines between subjects with high versus low activity that was derived from the analyses on of the baseline assessment of the same study sample (1, 2). However, this negative association included both cytokines with predominately pro-and anti-inflammatory properties and thus, our results do not portend that this immune-modulation is specifically anti-inflammatory. Training interventions also did not consistently show anti-inflammatory effects but rather a reduction in both anti-and pro-inflammatory cytokines (34). The effects of physical activity on the cytokine-modulating effects may result from its positive effect on the amount visceral adiposity as well as on the degree of adipose tissue inflammation, an increase of regulatory T cells in the systemic circulation, an improvement of the gut barrier function resulting in reduced endotoxaemia as well as the reduction of inflammation-associated comorbidities (17). In obesity, physical activity can decrease both the systemic and local synthesis and release of cytokines by inducing fatty acid oxidation of visceral adipose tissue, by reducing the immune cell infiltration in the adipose tissue and by reducing insulin resistance which prevents the migration of M1-macrophages (17, 35, 36). Further, training may increase the release of transcriptional factors (e.g. PGC1α) whose gene expression positively correlates with the reduction of pro-inflammatory proteins, such as IL-6 and TNF-α (37). On the other hand, the longitudinal observation could not reveal an association between changes in activity and cytokines. This may be due to the fact that the observational period did not go along with a training program whose intensity seems to be associated with the changes in cytokines (38, 39). Since the effect of training may mediate its effect *via* a reduction in adipose tissue, the missing changes in the BMI during the two points of measurement may further account for this missing association.

An association between the amount of adipose tissue and pro-inflammatory cytokine levels has been reported earlier (5, 40). We could confirm such an association at both time points. However, although there may be an association between the body composition and the cytokine profile, the backward regression analyses revealed that the BMI could only explain the levels of IL-12 and IL-13 at T2 in this group of obese and non-obese subjects. Since our previous investigation has shown that the association with anthropometrics is pronounced in samples of obese subjects and only to a lesser extend in non-obese subjects (1), we may conclude that the mix in subjects with and without obesity may, in part, account for the missing explanatory value of the BMI. Further, the assumption that obesity is simply related to a pro-inflammatory state needs to be questioned since the levels of anti-inflammatory cytokines were equally related to the BMI and weight. The increase in anti-inflammatory IL-10 with rising BMI for example may serve as a counteraction to the pro-inflammatory state, as IL-10 down-regulates TNF, IL-12 and GM-CSF (41).

Concerning the link between mood states and cytokines, the findings between the two times of measures are inconsistent. Since the BDI2, as well as the levels of cytokines, did not change relevantly between the two time points and we could not identify other factors that may have impeded the results, this connection, at least in mentally healthy subjects, has to remain open for debate. It needs to be kept in mind that the subjects included in this analysis did not suffer from a major depression but showed no or only little symptom load and for whom the plain BDI2 sum score may not be the suitable indicator. For community-dwelled samples without a clinical depression, previous results on the association between depressive symptoms and cytokine levels were also both positive (42) and negative (43). Potentially, it needs a certain threshold or symptom load of depression, which our sample did not exhibit, that leads to an immune activation, as a dependency of the cytokine levels from the depression severity and state has been found (44). Further, it may need a stimulation of the cytokine expression to correspond with the clinical picture (45), which the subjects in this sample did not experience. In clinical depression, findings were also disparate with a positive, negative or no relationship in depression (11, 13, 46). For clinical depression, which could also hold true for sub-clinical mood states, researchers have proposed a relationship between cytokines and certain symptoms or sub-groups of depression rather than a severity-dependence (12, 14, 42, 47).

Cytokines have previously been described to have sleep- or wakefulness-promoting effects (20, 21, 48), to relate to the presence of daily naps or daytime sleepiness (1, 20) and were seen to relate to the cerebral wakefulness regulation, i.e. the brain arousal (26). However, in this investigation in which the duration of sleep was measured objectively by actigraphy, as well as others (49), no such observation could consistently be found, which has to question this relationship.

A number of limitations have to be considered when interpreting our results: As a consequence of the applied quality criteria for our analysis, especially with regard to the actigraphy, the number of subjects included into the final analyses was relatively small to fully rule out type-II-errors, impeded sub-group-analyses as well as the inclusion of further parameters. The observational period varied between the subjects, which could interact with the results at T2 and the dynamics between the time points. Further, the subjects did not undergo a systematic interventional program and, thus, the changes in the psycho-biological parameters between the two time points varied distinctly. A group undergoing interventional programs concerning the factors could have added valuable information to the findings in this observational setting.

In conclusion, this investigation on the association between physical activity, subjective well-being, sleep parameters and body composition and inflammatory mediators revealed that predominately, the degree of physical activity is associated with certain cytokines. This could be relevant when focusing on anti-inflammatory treatment strategies. Since the results differed between the two assessments, although no specific intervention, but rather clinical management including a range of therapies in some patients had been performed in between, the conclusions for an association between the parameters cannot be considered as definite but give good reason for further research on this highly relevant field.

## Data Availability Statement

The datasets generated for this study are available on request to the corresponding author.

## Ethics Statement

The studies involving human participants were reviewed and approved by Leipzig University Ethics Committee. The patients/participants provided their written informed consent to participate in this study.

## Author Contributions

FS and CS designed the study. JM, FS, and HH recruited the participants. FS, CS, RM, and HH wrote the manuscript. LH and DT conducted the chemical analyses. CS, RM, and FS performed the statistical analyses. JM, RM, and CS revised the manuscript. All authors contributed to the article and approved the submitted version.

## Funding

This work was supported by the Federal Ministry of Education and Research (BMBF), Germany, FKZ: 01EO1001. This study was supported by the Integrated Research and Treatment Centre for Adiposity Diseases (IFB), University of Leipzig which is funded by the Federal Ministry of Education and Research (BMBF; Grant Number: 01EO1001). The authors acknowledge support from the German Research Foundation (DFG) and Universität Leipzig within the program of Open Access Publishing. HH has received salary support from the National Institute for Health Research (NIHR) Biomedical Research Centre (BRC) at South London and Maudsley NHS Foundation Trust (SLaM) and King’s College London. The funding sources had no role in the design and conduct of the study; collection, management, analysis, and interpretation of the data; and preparation, review, or approval of the manuscript.

## Conflict of Interest

The authors declare that the research was conducted in the absence of any commercial or financial relationships that could be construed as a potential conflict of interest.
